# A pilot study of the duration of GP consultations in Ireland

**DOI:** 10.1186/s40814-019-0532-4

**Published:** 2019-12-01

**Authors:** Tom Pierse, Luke Barry, Liam Glynn, Diarmuid Quinlan, Andrew Murphy, Ciaran O’Neill

**Affiliations:** 1 Health Economic and Policy Analysis Centre, National University of Ireland Galway, Galway, Ireland; 2 Graduate Entry Medical School and Health Research Institute, University of Limerick, Limerick, Ireland; 3 Department of General Practice, University College Cork, Cork, Ireland; 4 Discipline of General Practice, School of Medicine, National University of Ireland Galway, Galway, Ireland; 5 Centre for Public Health, Queens University Belfast, Belfast, UK

**Keywords:** GP, Consultation duration, Consultation time, GP workload, Primary care

## Abstract

**Background:**

General practitioner (GP)-led primary care is the linchpin of health care in Ireland. Reflecting international trends, there are increasing concerns about the sustainability of the current Irish GP service due to an increasing workload. Objective data on the duration of GP consultations are currently not available in Ireland. The objective of this pilot study is to demonstrate how the duration of consultations can be collected, using readily available administrative data.

**Methods:**

Software was developed to extract the duration of GP consultations using the opening and closing of electronic patient records associated with a GP consultation. GP practices (*N* = 3) comprising 15 GPs were recruited from a university-affiliated research network. A retrospective analysis of GP consultations with patients with diabetes for the 9 years between 2010 and 2018 was used to assess the feasibility of using this system to measure the duration of consultations.

**Results:**

The average duration of a consultation was 14.1 min for the 9 years spanning 2010 to 2018. Patients had an average time between consultations of 99 days.

**Conclusions:**

This pilot study confirms that an administrative data set can be utilised at negligible cost to monitor GP practice consultation workload over time. Our preliminary pilot data show that GP consultation durations among participating practices were longer than the 5–11.7 min reported in the UK and show an increase over the period. Clearly, a larger number of practices and patients are required to substantiate this finding.

## Background

Population growth, policy measures that have widened access, and population ageing with increasing multi-morbidity have contributed to an increase in demand for GP services in Ireland [1]. This has coincided with an increase in emigration among newly trained GPs, an increase in part time working by many existing GPs [2] and many GPs nearing retirement age [3]. In the absence of increased training and recruitment, these trends will place increasing pressure on those who remain to deliver an increasingly complicated and administratively demanding service [4].

The duration of GP consultations varies widely across Europe; in the UK, the average consultation duration has been variously reported at 5 to 11.7 min whereas in Sweden the average duration is 22 min [5, 6]. Trends in consultation duration are available for a small number of countries; these indicate stable or slowly increasing consultation durations [5]. Currently, the only estimates of GP consultation durations in Ireland are based on GP recall [2]. In a survey of 462 randomly selected GPs, respondents stated the number of face-to-face consultations that they would complete in a single clinical session [2]. The majority of GPs (64%) reported seeing 15 patients per session, which suggests 12 min per consultation if a 3-h session is assumed [2].

Consultation durations have been shown to be related to the characteristics of the patient, the practice, the practitioner, and the consultation [7–9]. Important patient characteristics related to increased consultation length include the degree of multi-morbidity and to a lesser extent patient age [7, 9–11]. Practice characteristics related to the duration of consultations include the practice workload [11]. Practitioner characteristics associated with the duration of consultations include gender and experience. Orton and Pereira [12], for example, found that female GPs have consultations that are longer than their male counterparts and that doctors registered more than 10 years have shorter consultations than those registered less than 10 years.

While there is a paucity of high-quality evidence on the effects of changing the duration of consultations on outcomes, there are indications of improved quality of care associated with longer consultation durations [5, 13, 14]. Increased consultation durations are associated with increased patient enablement, more accurate diagnosis of psychological problems [14], more preventative care [15], and reduced hospital admissions for some conditions [5]. Increased consultation durations have also been shown to be related to reduced GP stress and burnout [5, 10].

There is sparse evidence around the relationship between the length of GP consultations and patient satisfaction [13, 16]. There is also little evidence on the extent to which consultation durations are affected by the relationship between the GP and the patient. One study found that patients that were new to a practice had significantly longer consultations [9].

What is abundantly clear is that there is a paucity of evidence based on the use of reliable data collected at scale on the duration of consultations in Ireland. Lacking such evidence, it is challenging to identify if, and why these have changed over time, or what effect this may have had on patient outcomes and satisfaction. The aim of this study was to demonstrate, as a proof of principle, how consultation duration data could be readily extracted from software used routinely by Irish GPs. We then outline multiple potential applications of this data source for health service improvement, planning, and research in Ireland. As defined by Eldridge et al. [17], this is a pilot study intended to support the conduct of a later and much larger study.

## Methods

### Practice selection and data extraction

GP practices using the Socrates practice management software were recruited from a university-affiliated research network. Information was sent to practices inviting them to participate in this study, outlining the study aims and how to participate. Participating practices were asked to ensure all practitioners consistently opened and closed patient records before and after each consultation over a 2-week period in 2018. Participating practices also undertook a software upgrade to enable extraction of study data. As this was a pilot study, it was decided to collect data from three practices. The data from the GP practice software was extracted and anonymised by the Irish College of General Practitioners (ICGP). All data collected was completely anonymised to the researchers.

### GP information technology

In Ireland, there are four GP practice management software packages accredited by the Irish College of General Practitioners [18]. The Socrates GP software package is one of these and used by 17.3% of GPs in the West of Ireland [19]. The Socrates system captures information entered during a consultation, including test results, referrals, and diagnoses. The duration of consultations is captured by recording the time the patient record is opened at the start of a consultation and closed at the conclusion. This offers a platform through which the duration of consultations may be identified and captured.

### Data capture in the GP practice

The software to capture the duration of consultations was developed as part of an unrelated diabetes study. The software harvested duration of consultation data solely for those patients with diabetes. The duration of a consultation was only included when the patients’ file was accessed for a clinical consultation with a GP; patient files opened for administrative purposes are identifiable in the database and were excluded from the analysis. The duration of a consultation was linked to the patient’s data using a unique identifier. The software rounded the duration of consultations to the nearest minute. Data were captured from January 2010 (2 practices) and November 2010 (1 practice) to January 2019 (all practices).

### Data validation

The accuracy of the duration of consultation data depends upon the patient’s record being opened at the beginning and closed at the end of each consultation in a consistent fashion. A GP may keep a patient’s clinical file open once the consultation is finished, resulting in erroneously long durations. To assess the extent to which this might occur, a validation exercise was undertaken. In this, participating GPs were explicitly asked to close each patient’s file at the end of the consultation during a 2-week period from 28 November to 11 December 2018. This period was compared to the 2-week period prior to the explicit request (13 November to 27 November 2018). The difference in the mean duration between the two periods was calculated to identify any change in patterns of duration between the two periods.

### Statistical comparison of durations

A log-linear model was used to compare the duration of consultations across GP practices due to right-skewed distribution of the duration variable.

## Results

Data on the duration of 13,786 consultations involving 577 individual patients were extracted as part of our study. Complete consultation duration data were available from January 2010 (2 practices) and November 2010 (1 practice) to January 2019 (all practices). The unit of observation of the data is the practice—individual GPs are not identified. The records of consultations are for normal working hours, Monday to Friday.

The characteristics of the participating practices and patients are shown in Table 1. While the patient characteristics data is longitudinal, the mean age, gender, diabetes type, and patient type (public or private) are broadly in line with a previous cross-sectional study of diabetes in Ireland which report both type I and type II diabetes as well as gestational diabetes [20]. Any differences may be a reflection of differences in consultation frequency and the inclusion of different types of diabetes.

**Table 1:** Patient and practice characteristics

Patient characteristics	
Age years (mean)	63.4
Men (%)	56.0
Diabetes type (%)	
Gestational	5.7
Type 1	12.9
Type 2	81.4
Patient type (%)	
Public	65.0
Private	35.0
Practice characteristics	
Number of practices	3
Rural (%)	66.6
Total patients in all practices	41,254
Public patients (%)	47.8
No. of GPs	15
No. of practice nurses	3
GP age (mean)	46
Female GP (%)	70.2

**Fig. 1:**
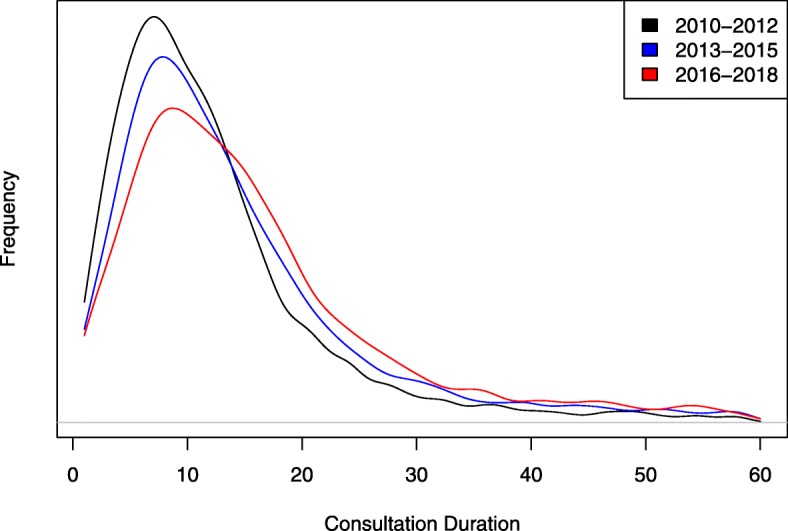
Kernel density plot of the duration of consultations for three time periods

The mean consultation duration was 14.1 min (SD = 10.6), and the mean time between consultations was 99 days. To visualise the distribution of consultation durations and changes in the distribution over time, we split the data into three time periods, shown in Fig. 1. The distribution shifts to the right over time indicating a trend toward longer duration of consultations. As shown in Table 2, two of the three practices have had statistically significant increases in consultation durations between the first and third time periods.

**Table 2:** Duration of consultation by practice for three time periods

	P1:2010–2012, mean (S.D.)	P2:2013–2015, mean (S.D.)	P3:2016–2018, mean (S.D.)	Diff P1:P3, *p* value
Practice A	17.0 (10.8)	19.5 (11.9)	19.1 (11.9)	0.01
Practice B	13.3 (10.1)	12.8 (9.6)	13.8 (9.6)	0.27
Practice C	9.4 (6.7)	11.6 (9.5)	15.0 (11.7)	< 0.001

### Validation exercise

The validation exercise carried out involved comparing the mean duration of consultations in the period prior to the explicit request that consultation files be closed on termination of the consultation, from 13 to 27 November 2018, with the period from 28 to 11 December 2018. There were a total of 76 and 79 consultations with people with diabetes in each of these periods respectively. A comparison of these two periods shows no change in the mean consultation length (14.99 min vs 14.86 min, *p* value 0.93).

## Discussion

This pilot study confirms that Irish GP consultation workload has the potential to be objectively measured over time, using an easily accessible, low-cost data source. There are potentially a wide range of uses for such data. These include monitoring trends in average durations over time to show the effects of policy interventions on GP utilisation, identifying capacity constraints across the country and the impact of these on consultation times, and assisting GPs to benchmark against each other. With a fuller set of practice records from a large nationwide sample, for all patients and not just those with diabetes, it would be possible to more fully explore these questions. This study though provides a proof of principle with respect to the feasibility of the data collection process.

The usefulness of such data in assessing the impact of policy changes, such as the introduction of universal health care, is self-evident. As we have demonstrated, the data is available as a time series in what appears to be a reliable format that allows the number and duration of each consultation to be measured. While we have only explored the use of the system with respect to patients with diabetes, it could be easily expanded to other patient groups and used to explore the impact of initiatives here. For example, it is proposed to expand the availability of free GP care to all those aged under 12 years in Ireland [21]. The data could also be used to estimate the cost of a GP consultation for use in health technology assessments and indeed bring greater granularity to this in terms of different patient types than is available in UK sources such as the PSSRU Unit Costs [22].

The data would also be useful in relating the duration of consultations with the frequency of consultations [5]. Policies, such as structured diabetes care, addressing a range of issues during a single consultation, will require protected clinical time. Previous studies have found that GP consultation durations may be increased by 2 min for each additional presenting problem [23]. It remains to be seen whether such extended consultations will reduce the frequency of GP visits.

While only three practices were included in our study and while it was not the focus of our attention, it is interesting to note that despite increased demands on GP time, consultation durations increased in two out of the three practices over the period. Whether this is typical of other patient groups or other GP practices is unclear.

### Limitations

The data presented in this study are from 3 GP practices with 15 GPs, in a university-affiliated research network, and may not be representative of mainstream Irish GP practice. The duration of consultation data collected in this pilot was solely for patients with diabetes, who are not representative of all patients. This clearly limits a broader comparison of the GP duration of consultations, but the proof of concept emerges intact. Similar data could readily be extracted for other patients, enabling comparisons. Similarly, while we extracted data using an add-on to the Socrates software package, the authors have confirmed with the ICGP that similar arrangements could be readily implemented in other software platforms.

Future studies attempting to use this data may wish to implement further validation exercises. Information was provided to practices requesting patient files be closed directly after the end of the consultation. While this exercise showed that there was no difference between the periods before and after the explicit request was made, it does not confirm that GPs are using the software in the desired manner. Therefore, there is a risk that consultation durations may be overestimated. This can only be confirmed by comprehensive ‘time and motion’ research.

Much of the work on consultation durations have focused on the GP consultation. The practice nurse undertakes a quarter of consultation in UK general practice [7]. We do not observe in the current data whether the consultation is nurse or GP provided. This would be a useful addition to the software and has been collected in similar international studies [6, 7].

## Conclusions

In this study, we have demonstrated that it is feasible to collect the duration of consultations in Ireland, at a negligible cost using electronic administrative records. We outline the multiplicity of applications for such data including tracking the consultation workload of GPs over time. This is important in the context of increasing demands on GP time due to increasing population, increasing multi-morbidity and policy changes such as the move toward universal health care.

## Data Availability

The data that support the findings of this study are available from the ICGP, but restrictions apply to the availability of these data, which were used under licence for the current study, and so are not publicly available.

## Abbreviations

GP: General practitioner
ICGP: Irish College of General Practitioners
